# Cross-regulation between SOX9 and the canonical Wnt signalling pathway in stem cells

**DOI:** 10.3389/fmolb.2023.1250530

**Published:** 2023-08-17

**Authors:** Jiajia Wang, Xichen Wan, Qihua Le

**Affiliations:** ^1^ Department of Ophthalmology, Eye, Ear, Nose, and Throat Hospital of Fudan University, Shanghai, China; ^2^ Research Center, Eye, Ear, Nose, and Throat Hospital of Fudan University, Shanghai, China; ^3^ Myopia Key Laboratory of Ministry of Health, Eye, Ear, Nose, and Throat Hospital of Fudan University, Shanghai, China

**Keywords:** SOX9 transcription factor, Wnt signalling pathway, crosstalk, signalling transduction, stem cell

## Abstract

SOX9, a member of the SRY-related HMG-box transcription factors, has been reported to critically regulate fetal development and stem cell homeostasis. Wnt signalling is a highly conserved signalling pathway that controls stem cell fate decision and stemness maintenance throughout embryonic development and adult life. Many studies have shown that the interactions between SOX9 and the canonical Wnt signalling pathway are involved in many of the physiological and pathological processes of stem cells, including organ development, the proliferation, differentiation and stemness maintenance of stem cells, and tumorigenesis. In this review, we summarize the already-known molecular mechanism of cross-interactions between SOX9 and the canonical Wnt signalling pathway, outline its regulatory effects on the maintenance of homeostasis in different types of stem cells, and explore its potential in translational stem cell therapy.

## 1 Introduction

*SRY*, a sex-determining gene, was first found in human and mouse Y-chromosomes in 1990 (Gubbay et al., 1990; Sinclair et al., 1990). The SOX family is a group of transcription factors that contain the DNA-binding domain of the SRY-related high-mobility group (HMG) box (Moradi et al., 2017). The HMG domain contains 79 amino acids and binds with the minor groove of special DNA with consensus sequences (A/TA/TCAAA/TG) (Jo et al., 2014). Over the last several decades, more than 20 *SOX* genes have been found based on the homology analysis of highly conserved HMG boxes, and they have been further classified into eight subgroups (SOX A-H) (Bowles et al., 2000; Schepers et al., 2002).

**FIGURE 1:**
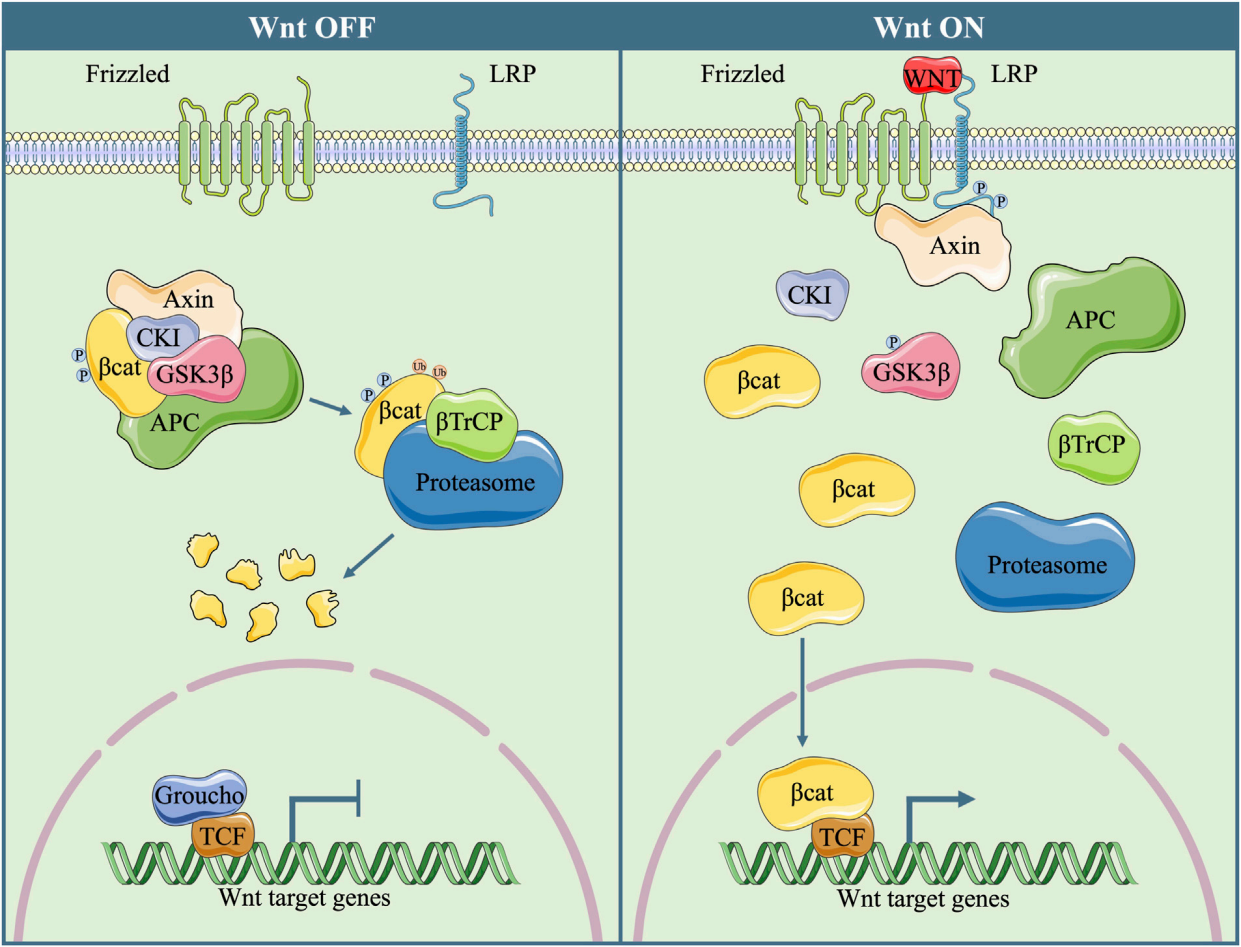
Diagram of the canonical Wnt Signalling Pathway In the absence of WNT proteins, a destruction complex, which is composed of Axin, APC, CKI and GSK3β, phosphorylates β-catenin and induces its degradation in a ubiquitination/proteasome-dependent manner. Without the presence of nuclear β-catenin, TCF combines with co-repressor Groucho, and the transcription of downstream target genes is suppressed. When WNT protein is present, phosphorylated LRP causes decomposition of the destruction complex and GSK3β inactivation. Then, dephosphorylated β-catenin translocates into nucleus, combines with TCF, and initiates the transcription of downstream target genes.

WNT is a ligand family that is composed of 19 proteins. In mammalian cells, the Wnt signalling pathway plays a critical role in the self-renewal and fate determination of stem cells (Nakatsu et al., 2011), as well as tumorigenesis and metastasis (Sugimura and Li, 2010). WNT proteins activate the transcription of target genes either in a β-catenin-dependent pathway (canonical pathway) or through a β-catenin-independent cascade (noncanonical pathway). In the absence of WNT ligands, a destruction complex that is composed of adenomatous polyposis coli (APC), the scaffolding protein AXIN, casein kinase I alpha (CKIα), and glycogen synthase kinase 3 beta (GSK3β) causes the phosphorylation and ubiquitylation of β-catenin, ending up with its proteasome-dependent degradation and the inactivation of the canonical pathway (Nusse and Clevers, 2017; Rim et al., 2022; Shah and Kazi, 2022). However, when WNT proteins are present, they combine with frizzled-class receptors (FZDs) and two low-density-lipoprotein-receptor-related protein (LRP5/6) co-receptors, inhibit the activity of the destruction complex, and increase the stability of β-catenin. Then, β-catenin translocates into the cell nucleus, interacts with other transcriptional factors, such as the T-cell factor/lymphoid enhancer factor (TCF/LEF) transcription factors (Eastman and Grosschedl, 1999), and induces the transcription of downstream target genes (Figure 1).

It has been reported that both the canonical and noncanonical Wnt signalling pathways have interactions with the SOX family, which participates in various physiological activities and pathological conditions of stem/progenitor cells (Moradi et al., 2017). For example, SOX5 inhibits the transcriptional activity of β-catenin and regulates the cell cycle in neural progenitors (Martinez-Morales et al., 2010). WNT7b enhances the self-renewal and osteogenic differentiation of bone marrow mesenchymal stem cells by activating the Ca^2+^/NFATC1 signalling pathway and inducing elevated expression of SOX11 (Yu et al., 2020).

SOX9, a member of the SOXE family, is an important transcription factor involved in sexual determination, stem cell development, and tumorigenesis (Bastide et al., 2007; Sellak et al., 2012; She and Yang, 2015). In recent years, there has been a growing number of studies reporting that SOX9 has complicated interactions with the Wnt signalling pathway. In this narrative review, we summarize the molecular mechanism of the interactions between the SOX9 transcription factor and the canonical Wnt signalling pathway. The effect of the SOX9-Wnt axis on the development and homeostasis maintenance of stem cells is also reviewed so as to explore its potential in translational stem cell therapy.

## 2 Structure of the SOX9 protein

**FIGURE 2:**
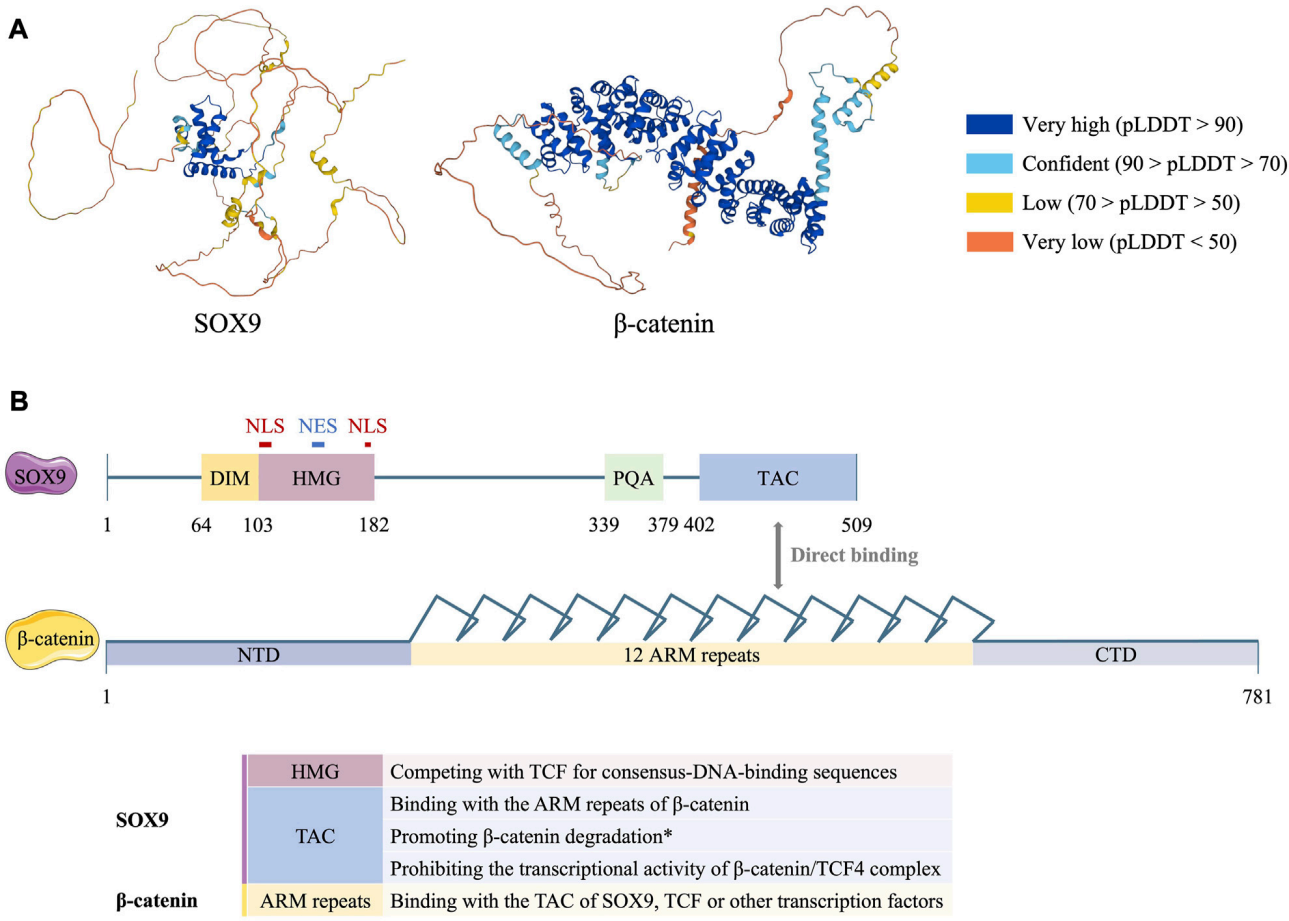
Protein structure of human SOX9 and β-catenin **(A)** Predicted 3D structure of human SOX9 and β-catenin proteins (produced by Software AlphaFold (Jumper et al., 2021; Varadi et al., 2022)). pLDDT refers to per-residue confidence score between 0 and 100. The dark blue portion of SOX9 with very high pLDDT corresponds to its HMG domain, while the dark blue portion of β-catenin corresponds to its 12 ARM repeats. **(B)** Functional domains of human SOX9 and β-catenin protein. Numbers refer to amino acid residues. ARM, armadillo; CTD, C-terminal domain; DIM, dimerization domain; HMG, high-mobility group; NES, nuclear export signal; NLS, nucleus localization signal; NTD, N-terminal domain; PQA, proline-glutamine-alanine-rich; TAC, C-terminal transactivation domain. *Controversially.

Like other members of SOX family, the SOX9 protein is characterized by an SRY-related HMG domain that has three α-helices (Figure 2A) with 50% amino acid similarity (Kamachi and Kondoh, 2013). The HMG domain of SOX9 contains two independent nucleus localization signal (NLS) sequences and one nuclear export signal (NES) sequence (Jana et al., 2020), which determine the location of the SOX9 transcription factor in either the cell nucleus or cytoplasm. Moreover, the N-terminal dimerization domain (DIM) facilitates the homologous dimerization of two SOX proteins, while the C-terminal transactivation domain (TAC) promotes the interaction of SOX9 with coactivators or other transcription factors (Figure 2B) (Huang et al., 2015; She and Yang, 2015). Additionally, a proline-glutamine-alanine (PQA)-rich motif, which maps to residues 340–379 of SOX9, enhances the transactivation potency of TAC (Barrionuevo and Scherer, 2010).

## 3 Molecular mechanism of cross-regulation between SOX9 and the canonical Wnt signalling pathway

**FIGURE 3:**
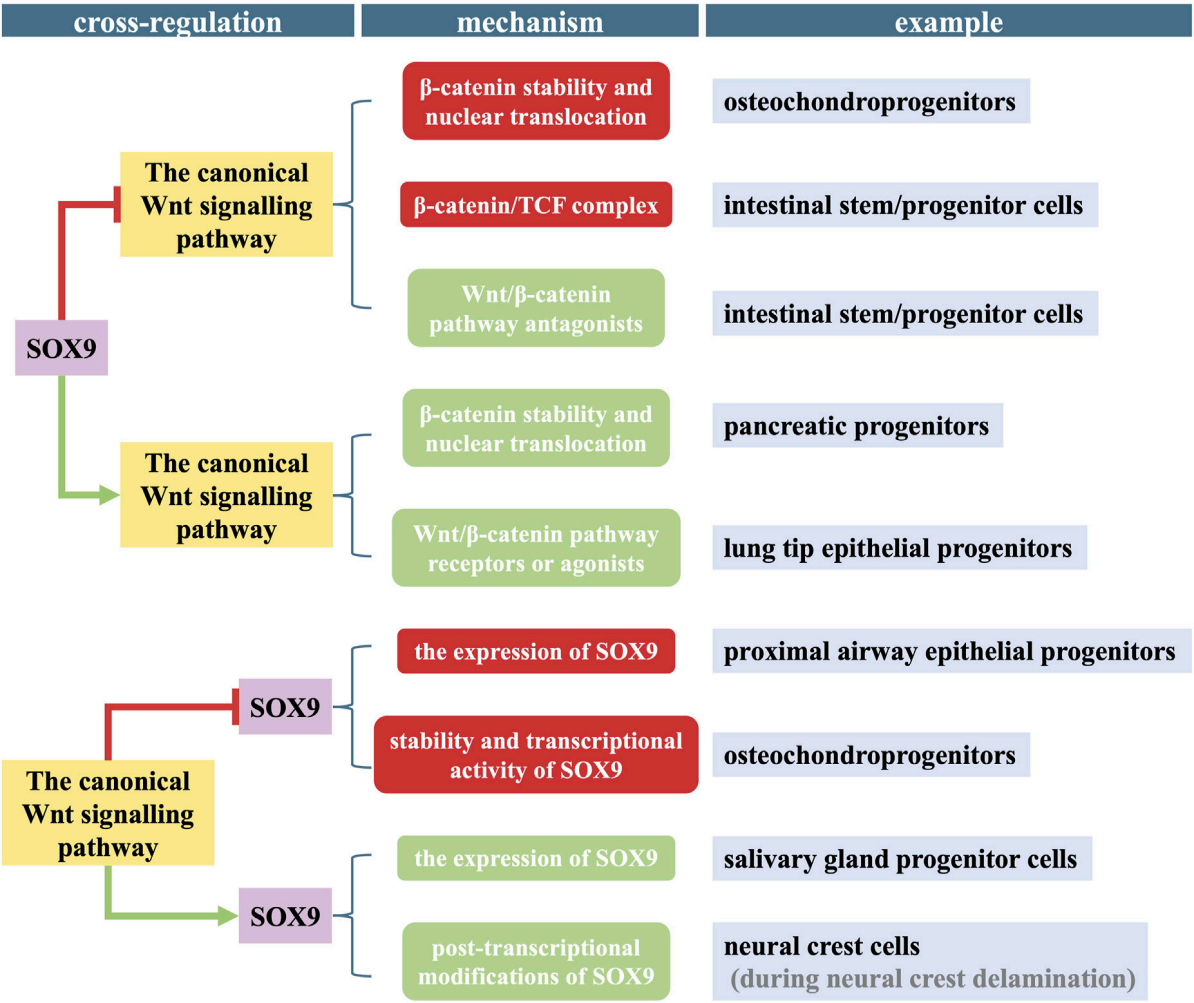
Diagram of the cross-regulation between SOX9 and the canonical Wnt pathway in different stem/progenitor cells. The molecular mechanism of their mutual antagonism or enhancement is complicated and involved in different types of stem/progenitor cells and/or physiological activities. Post-transcriptional modification includes phosphorylation and SUMOylation. Red block signs and rectangles refer to INHIBITION, green arrows and rectangles refer to ACTIVATION.

SOX9 and the canonical Wnt pathway have complicated interactions and cross-regulation (Figure 3). Their mutual antagonism or enhancement has been reported in different types of stem cells and developmental phases, and they form a subtle balance to maintain normal physiological activities.

### 3.1 SOX9 represses the canonical Wnt signalling pathway

**FIGURE 4:**
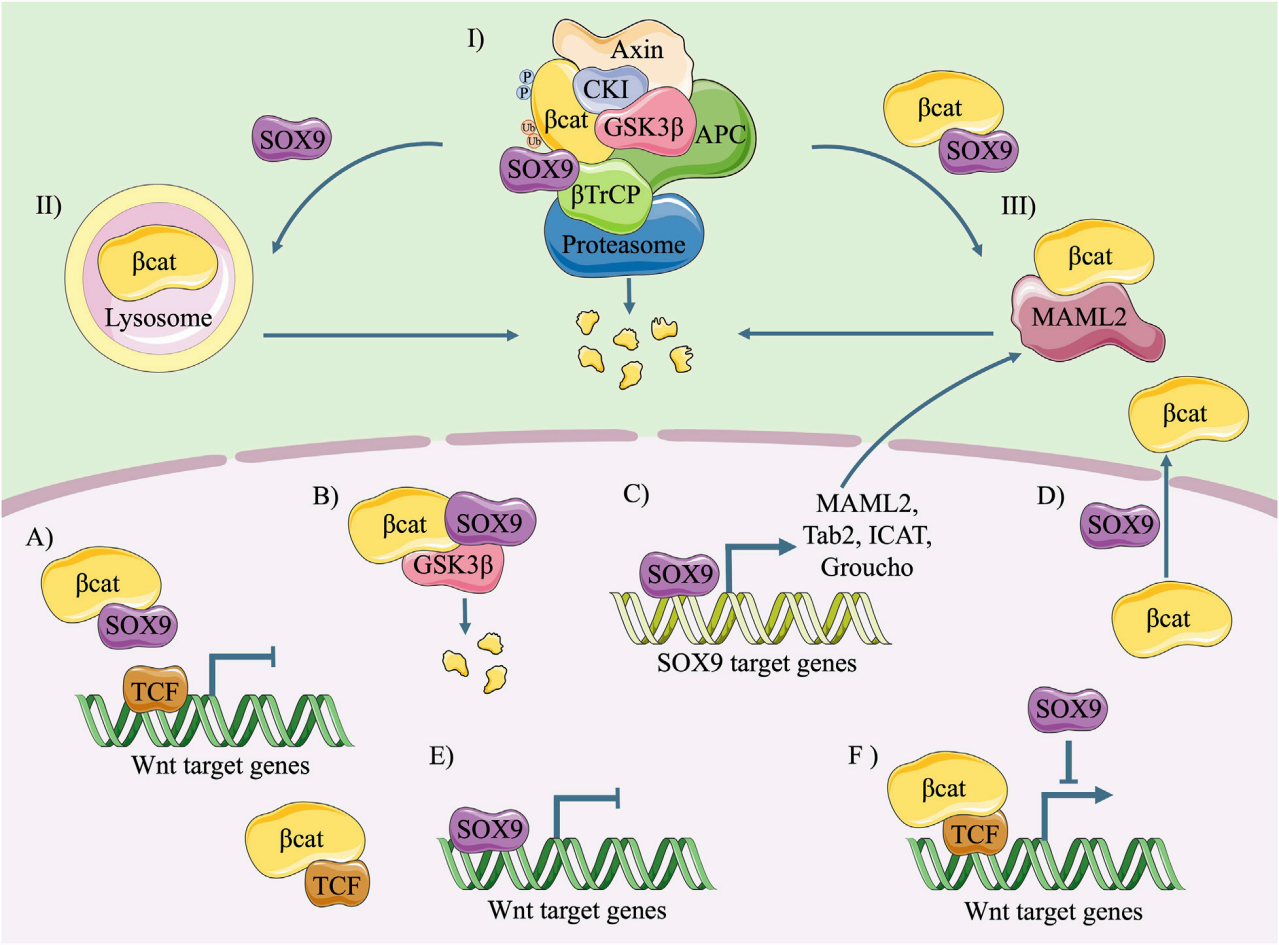
Diagram of the molecular mechanism of SOX9 inhibiting the canonical Wnt pathway. In the cytoplasm, SOX9 induces degradation of β-catenin via ubiquitination/proteasome **(I)**, lysosome **(II)** or MAML2 **(III)**. In the nucleus, SOX9 inhibits the canonical Wnt pathway through following ways: competitive binding with β-catenin and prohibited formation of β-catenin/TCF complex **(A)**; GSK3β (translocated by SOX9)-mediated β-catenin degradation **(B)**; promoting the expression of Wnt/β-catenin signalling antagonists **(C)**; inducing the re-localization of β-catenin from nucleus to cytoplasm **(D)**; replacing β-catenin/TCF complex and competitive binding with Wnt target genes **(E)**; and inhibiting the transcriptional activity of β-catenin/TCF complex **(F)**.

SOX9 is an important antagonist of the canonical Wnt signalling pathway (Figure 4). Nevertheless, the underlying mechanism has not been clearly addressed. Its molecular mechanism is possibly involved in three aspects: promoting the degradation of β-catenin, inhibiting the formation of a β-catenin-TCF/LEF complex and prohibiting its transcriptional activity, and transcriptionally activating Wnt-related antagonists.

#### 3.1.1 SOX9 impairs the stability of β-catenin and its nuclear translocation

SOX9 prohibits the activity of β-catenin in four ways: ubiquitination/proteasome-dependent degradation, lysosomal breakdown, mastermind-like transcriptional coactivator 2 (MAML2)-related turnover, and a reduced nuclear translocation level of β-catenin.

It has been reported that direct binding of β-catenin with the C-terminus of SOX9 (Figure 2B) results in the degradation of β-catenin in a ubiquitination/26S proteasome-dependent way (Figure 4Ⅰ) (Akiyama et al., 2004). Nevertheless, the indispensability of the C-terminus remains controversial because comparisons of the ability to induce β-catenin degradation between the SOX9 C-terminal deleted mutant (SOX9 △C) and full-length SOX9 had conflicting results (Akiyama et al., 2004; Topol et al., 2009; Au et al., 2023). Moreover, SOX9 induces the proteasome-dependent degradation of β-catenin in the cell nucleus. The N-terminal of SOX9 including the HMG domain is capable of inducing the nuclear translocalization of GSK3β and promoting its binding with β-catenin, thus leading to the phosphorylation and degradation of β-catenin in the nucleus (Figure 4B) (Topol et al., 2009).

Apart from ubiquitination/proteasome-dependent degradation, SOX9 has the ability to impair the stability of β-catenin in a lysosome-dependent way (Figure 4Ⅱ). After transfection with tagged SOX9, the stability of total β-catenin in HEK293 cells is maintained by lysosome inhibitor (NH4Cl) rather than proteasome inhibitor (MG132) (Cheng and Genever, 2010).

It should be noted that SOX9 activates the transcription of MAML2 (Figure 4C), a type of Notch signalling coactivator and β-catenin antagonist (Sinha et al., 2021). MAML2-dependent β-catenin degradation is the predominant way of restraining the canonical Wnt signalling pathway in circumstances with high levels of SOX9 and WNT protein (Figure 4Ⅲ). In contrast, the destruction complex is the overwhelming means of β-catenin turnover in the cytoplasm when the levels of SOX9 and WNT protein are low.

In addition, SOX9 regulates the nucleocytoplasmic shuttling of β-catenin. It has been reported that SOX9 induces a re-localization of β-catenin from the nucleus to the cytoplasm (Figure 4D) in a colon cancer cell line (Prevostel et al., 2016). However, its underlying mechanism needs further study to be clearly addressed.

#### 3.1.2 SOX9 binds with β-catenin and prohibits the formation of a β-catenin/TCF complex and its transcriptional activity

The prevailing view is that SOX9 is able to directly bind with β-catenin and inhibit Wnt signalling (Akiyama et al., 2004; Abdel-Samad et al., 2011; Prevostel et al., 2016; Dash et al., 2021). β-catenin contains 781 amino acids, including a 130-amino-acid-long N-terminal domain (NTD), 12 ARM repeats, and a C-terminal domain (CTD) with 100 amino acids (Figure 2) (Lustig and Behrens, 2003; Shah and Kazi, 2022). The ARM repeats of β-catenin facilitate its binding with other transcription factors, such as TCF, in the nuclei (Bienz and Clevers, 2003). However, the TAC of SOX9 could compete with TCF/LEF and directly bind with the ARM repeats (Figure 2B), leading to the arrested formation of the β-catenin-TCF/LEF complex (Figure 4A) (Akiyama et al., 2004; Sellak et al., 2012). Moreover, a similar HMG domain that identified consensus-DNA-binding sequences was found in both SOX9 and TCF (Clevers and van de Wetering, 1997). As reported, SOX9 is capable of replacing the TCF/LEF-β-catenin complex, occupying the consensus-DNA-binding site of Wnt target genes, and prohibiting their transcription (Figure 4E) (Huang et al., 2010). Even after the successful binding of the β-catenin/TCF4 complex to the promoter of Wnt target genes, SOX9 could also prohibit its transcriptional activity (Figure 4F) (Akiyama et al., 2004; Blache et al., 2004). The C-terminus of SOX9 is indispensable for this inhibition (Bastide et al., 2007; Topol et al., 2009; Au et al., 2023).

#### 3.1.3 SOX9 promotes the expression of Wnt/β-catenin signalling antagonists

It has been reported that SOX9 increases the transcriptional activity of many inhibitory molecules of the canonical Wnt pathway (Figure 4C) (Kormish et al., 2010; She and Yang, 2015), such as Tab2 (Huang et al., 2017), MAML2 (Sinha et al., 2021), the inhibitor of β-catenin and Tcf (ICAT), and the Groucho-related (Gro/TLE/Grg) family (Bastide et al., 2007). Moreover, SOX9 itself could be the transcriptional inhibitor of cyclin-dependent kinase 1 (*CDK1*), one of the target genes of the Wnt signalling pathway (Huang et al., 2017).

### 3.2 SOX9 activates the canonical Wnt signalling pathway

**FIGURE 5:**
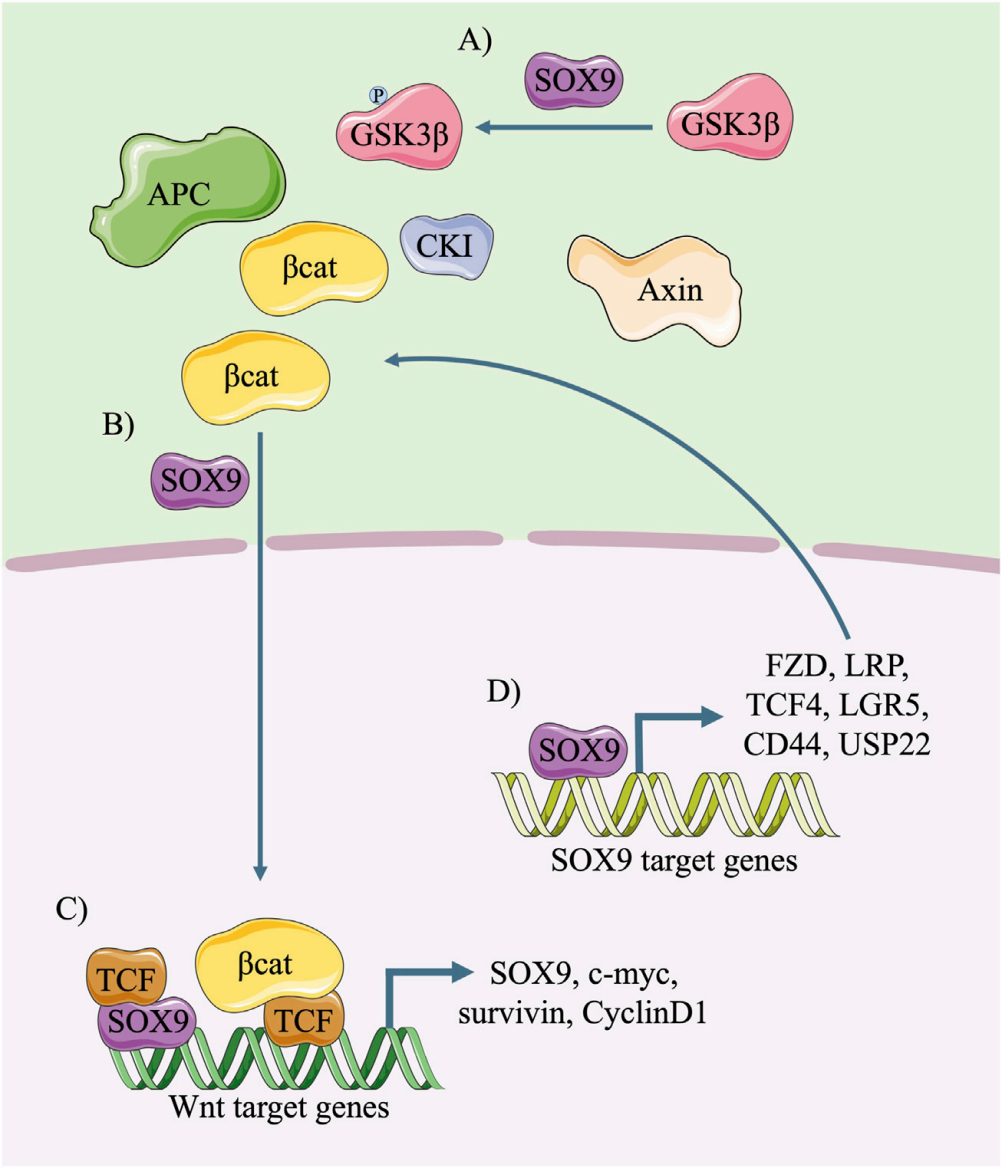
Diagram of mutual activation between SOX9 and the canonical Wnt pathway. In cytoplasm, SOX9 phosphorylates GSK3β, improves the stability of β-catenin **(A)** and promotes its nuclear translocation **(B)**. In the nucleus, the binding of SOX9/TCF complex to Wnt-responsive enhancer promotes the transcription of downstream molecules including SOX9 **(C)**. The transcription of Wnt signalling pathway components and amplifiers, such as FZD, LRP and TCF4, could be activated by SOX9 and further promotes the canonical Wnt/β-catenin signals **(D)**.

Many studies suggest that SOX9 has the potential to activate the canonical Wnt/β-catenin signalling pathway (Figure 5) in stem/progenitor cells or cancer cells. It has been proven that SOX9 activates the canonical Wnt signalling pathway predominantly by promoting the stability and nucleus translocation of β-catenin (Figures 5A, B) (Kormish et al., 2010; Kawai et al., 2016; Zhang et al., 2019; Chen et al., 2022; Li et al., 2023), in which the inactivation of GSK3β by SOX9 via the phosphorylation of Ser9, an increased level of β-catenin translocation into cell nuclei, and the enhanced transcriptional activity of the β-catenin/TCF complex are involved (Huang et al., 2019). Moreover, SOX9 could directly bind to the enhancer of Wnt target genes in conjugation with TCF and promote their transcription (Figure 5C) (Ramakrishnan et al., 2023).

It is also notable that SOX9 plays a critical role in promoting the transcription of receptors and co-receptors of the Wnt signalling pathway (Figure 5D), including FZDs, LRP family members and TCF4 (Wang et al., 2013; Leung et al., 2016; Ma et al., 2016). The findings of ChIP-sequencing demonstrated that SOX9 bound to the enhancers of *Fzd8* and *Sox4* and promoted their transcription in intestinal stem cells (ISCs) (Huang et al., 2017). In lung tip progenitors, SOX9 directly upregulated the transcription of LGR5 and CD44, which are the amplifiers of the Wnt signalling pathway (Sun et al., 2022). Moreover, SOX9 was also reported to bind the promoter of ubiquitin-specific peptidase 22 (USP22), a deubiquitinating enzyme, promote its transcription, and activate the Wnt/β-catenin pathway (Miao et al., 2022).

### 3.3 The Wnt signalling pathway regulates the expression of SOX9

The expression of SOX9 is regulated and maintained by the Wnt/β-catenin signalling pathway, as supported by many studies (Blache et al., 2004; Snowball et al., 2015; Liang et al., 2022; Liu et al., 2022; Sun et al., 2022). In tracheal cartilage and human lung bud tip progenitors, the elevation of either the WNT protein or Wnt signalling pathway amplifier enhanced the expression of SOX9 and maintained its level in the cell nucleus (Nasr et al., 2021; Hein et al., 2022). β-catenin participated in this process because it could partially rescue the loss of the SOX9 transcription and protein level that was caused by the absence of Wnt signalling activators (Sun et al., 2022). Meanwhile, the β-catenin/TCF4 complex could directly bind to the promoter of the *Sox9* gene and activate its transcription (Figure 5C) (Blache et al., 2004). Notably, post-transcriptional modifications are also involved in the regulation of the Wnt signalling pathway on SOX9. During neural crest delamination, the canonical Wnt signalling indirectly induces the phosphorylation of SOX9 on S64 and S181 and facilitates the modification of SOX9 by small ubiquitin-like modifier (SUMO) (Liu et al., 2013).

The Wnt/β-catenin pathway also plays an important role in inhibiting SOX9. It not only silences the *Sox9* gene via DNA methylation in limb bud mesenchymal cells (Kumar and Lassar, 2014), but also promotes the degradation of SOX9, which is predominantly in a ubiquitin/26S proteasome-dependent way (Akiyama et al., 2004; Jin et al., 2006). Microtubule-associated serine/threonine kinase 4 (Mast4), the stability of which is maintained by Wnt signals, might participate in this degradation (Kim et al., 2022). Moreover, β-catenin inhibits the transcriptional activity of SOX9 in a dose-dependent manner (Akiyama et al., 2004). However, the molecular mechanism of this inhibition remains unclear.

In summary, many parts of signalling transduction are involved in the cross-regulation between SOX9 and the canonical Wnt/β-catenin signalling pathway, including the expression and stability maintenance of key proteins, the nuclear translocation of signal molecules, the formation and activity of transcription complex, and selective downstream target genes, all of which form a complicated signal network. The effect of SOX9-Wnt cross-regulation and underlying mechanisms are tissue- and cell type-specific, and might have changes during the development or under pathological conditions, which will be addressed in details in the next section.

## 4 The role of SOX9-Wnt cross-regulation in the homeostatic maintenance of stem cells

Strong evidence has shown that the cross-talk between SOX9 and the Wnt signalling pathway regulates the delicate balance among stem cells, progenitor cells, and differentiated cells during development in normal tissues. Perturbation of such balances is associated with pathological conditions, such as dysplasia and cancer.

### 4.1 Airway progenitor cells

The effects of SOX9-Wnt interactions on airway progenitors are space-specific. For instance, the canonical Wnt/β-catenin signalling pathway is required to upregulate the expression of SOX9 and maintain the homeostasis of distal lung tip epithelial progenitors during cell differentiation and tissue development (Ustiyan et al., 2016; Ostrin et al., 2018; Hein et al., 2022; Sun et al., 2022). In contrast, β-catenin inhibits the expression of SOX9 in the progenitors of proximal airway epithelial cells (Ustiyan et al., 2016). The absence of β-catenin further leads to the expanded expression of SOX9 in the proximal airway (Rockich et al., 2013). These findings support the hypothesis that the space-specific control of SOX9 by β-catenin maintains a normal proximal–peripheral patterning of lung tubules during pulmonary branching morphogenesis (Ustiyan et al., 2016).

Moreover, during the development of the fetal lung, SOX9 amplifies Wnt signalling, promotes lung tip epithelial progenitor cell proliferation, and prohibits precocious airway differentiation (Sun et al., 2022). In the proximal airway, the reciprocal crosstalk between Wnt/Lef1 signalling pathway and SOX9 also dynamically controls the healing of superficial airway epithelium after injuries by regulating the asymmetrical division of submucosal gland progenitors, with one SOX9^+^Wnt^−^ daughter cell maintaining stemness and quiescence and the other SOX9^−^Wnt^+^ cell maintaining proliferation (Ievlev et al., 2022). Apart from its activity in epithelial cells, Wnt signalling is also required to activate SOX9 during the differentiation of tracheal mesenchymal stem cells and tracheal cartilage development (Snowball et al., 2015).

### 4.2 Pancreatic and intestinal progenitor cells

It was found that SOX9 phosphorylates GSK3β, upregulates the level of nuclear β-catenin, and promotes the proliferation and differentiation of pancreatic progenitors (McDonald et al., 2012). Another study indicated that miR-690 induces *Sox9* silencing and inactivates Wnt signalling, which leads to the arrested differentiation of β-cells from iPSC-derived insulin-producing cells (Xu et al., 2019). Nevertheless, the activation of Wnt signalling arrests the generation of β-cells by attenuating SOX9-dependent multipotent pancreatic progenitor cells (Dettmer et al., 2021). These findings collectively suggest that during SOX9-dependent pancreatic development and β-cell differentiation, the cross-regulation between SOX9 and Wnt signalling prevents the over-activation of SOX9. Intriguingly, the inactivation of SOX9 and a relatively low Wnt signalling level are crucial for the maintenance of functional adult β-cells, as evidenced by a study that found that aberrant activation of Wnt signalling and SOX9 in adult β-cells resulted in diabetes mellitus (Puri et al., 2013).

It has been demonstrated that the expression of SOX9 in the intestinal crypts, which is regulated by the Wnt signalling pathway, is indispensable for the development of Paneth cells, a type of highly specialized secretory epithelial cell that constructs the niches for ISCs (Blache et al., 2004; Bastide et al., 2007; Mori-Akiyama et al., 2007). Moreover, high-mobility group A1 (Hmga1) chromatin remodeling proteins upregulate Wnt agonist receptors and SOX9 and maintain ISC niches by expanding the Paneth cell compartments (Xian et al., 2017). Apart from its involvement with Paneth cells, SOX9 is also involved in the maintenance of an undifferentiated phenotype of intestinal epithelial progenitors in a Wnt-dependent manner (Blache et al., 2004). The molecular mechanism of the SOX9-Wnt axis in regulating the proliferation of ISCs was also explored. In diabetic mice, SOX9 transcriptionally activated the repressors of the canonical Wnt signalling pathway, such as Wnt4 and Tab2 (Huang et al., 2017). The absence of SOX9 led to the overactivation of the Wnt signalling pathway and abnormal proliferation and differentiation of ISCs. A bimodal role of SOX9 was proposed: A low level of Wnt-dependent SOX9 promoted ISC proliferation in the stem/progenitor cell compartment, whereas a high level of Wnt-independent SOX9 prohibited cell proliferation and induced terminal maturation of enteroendocrine precursors (Formeister et al., 2009).

### 4.3 Osteochondroprogenitors

The SOX9-Wnt signalling axis controls the lineage decisions of osteoblasts and chondrocytes (Akiyama et al., 2004; Hill et al., 2005). On one hand, the inactivation of Wnt/β-catenin signalling by SOX9 promotes cell differentiation from mesenchymal stem/progenitor cells (MSCs) into chondrocytes and the formation of cartilage nodules at the expense of osteogenesis (Hill et al., 2005; Topol et al., 2009; Dy et al., 2012; Venkatesan et al., 2012). On the other hand, the canonical Wnt/β-catenin signalling pathway represses the expression of SOX9, prohibits the chondrogenic potential of osteochondroprogenitors, and stimulates differentiation towards the osteoblast lineage (Hill et al., 2005; Kumar and Lassar, 2014; Lefebvre and Bhattaram, 2016). In addition, phase-specific mutual antagonism between SOX9 and β-catenin was found to be involved in the lineage decisions of osteochondroprogenitors. It was found that in D0 osteochondroprogenitors, SOX9 combined with the promoter of the target gene *Ccn2*, while β-catenin/TCF complex competed with SOX9 and bound to *Ccn2* in D26 hypertrophic chondrocytes (Huang et al., 2010). Such phase-specific regulations coordinate osteoblast recruitment, cartilage renewal, and bone formation.

However, reciprocal inhibition between SOX9 and Wnt/β-catenin signalling is disturbed in osteoblasts and chondrocytes under pathological conditions. Hydrostatic pressure induces the activation of Wnt/β-catenin signalling and, consequently, elevates the expression of SOX9 in MSCs, leading to a higher level of chondrogenic differentiation (Cheng et al., 2022). Nevertheless, during the induction of osteonecrosis of the femoral head (ONFH), the inhibition of SOX9 downregulates the Wnt/β-catenin signalling pathway and suppresses osteogenic differentiation (Meng and Zhu, 2023). It was also reported that the perturbation of mutual SOX9-Wnt inhibition contributed to dysplasia. In developing limbs, a truncated SOX9 mutant interfered with SOX9-mediated Wnt inhibition, resulting in campomelia, a kind of genetic disorder with skeletal malformation (Au et al., 2023). In addition, Wnt11, which instigates the noncanonical Wnt signalling pathway, was reported to promote the transcription of SOX9 and support the chondrogenic differentiation of MSCs (Liu et al., 2014).

### 4.4 Neural crest cells

Neural crest cells (NCCs) are a transient population of embryonic multipotent stem cells that give rise to a wide variety of cell and tissue types, including cartilage and bone, most neurons, and all glia of the peripheral nervous system (Achilleos and Trainor, 2012; Liu et al., 2013). Similarly to MSC-derived osteochondrogenesis, during NCC-derived craniofacial osteochondrogenesis, Wnt/β-catenin signalling enhances osteogenic potential by counteracting SOX9, and arrests cell differentiation towards chondrocytes in which Yap/Taz is involved (Dash and Trainor, 2020; Zhao et al., 2022). In addition, the absence of Med23 induces abnormally elevated expression of SOX9, which leads to the inhibition of Wnt signalling and the perturbation of NCC-derived mesenchymal proliferation in the palatal shelf (Dash et al., 2021).

Apart from their involvement in osteochondrogenesis, NCCs play a more important role in neural crest induction, which is mediated by Wnt signals, with SOX9 serving as a crucial downstream transcriptional activator (Lee et al., 2004). Yardley and Garcia-Castro (Yardley and Garcia-Castro, 2012) confirmed that the upregulation of WNT molecules is prior to the elevated expression of the neural crest marker, SOX9, during the transformation from non-neural ectoderm to the neural crest. Moreover, during the initiation of neural crest delamination, the canonical Wnt signalling promotes SOX9 phosphorylation and SUMOylation (Liu et al., 2013). SOX9 was also reported as a biomarker of adult neural-crest-derived stem cells, and it interacted with the canonical Wnt signalling pathway to maintain stemness (Hoving et al., 2021).

### 4.5 Limbal and follicle epithelial stem/progenitor cells

It was proposed that in the limbal niches, SOX9 and Wnt/β-catenin signalling mutually antagonize to achieve a balance among quiescence, proliferation, and differentiation of limbal epithelial stem/progenitor cells (LEPCs). In cultured LEPCs, *Sox9* knockdown caused a decreased level of GSK3β and increased expression of canonical-Wnt-signalling-related genes, such as *CTNNB1* (encoding β-catenin) and *WNT4*, which repressed cell proliferation and promoted differentiation (Menzel-Severing et al., 2018). In turn, the expression of SOX9 was significantly suppressed with the treatment of exogenous GSK3β inhibitors and was enhanced by C59, a small-molecule Wnt inhibitor. Likewise, an elevated level of Wnt3a, a Wnt ligand that only activates the canonical Wnt signalling pathway, downregulated the expression of SOX9 and impaired the stem cell phenotype (Peng et al., 2015). Our ongoing study also found out that Wnt16b, a ligand of both the canonical and the non-canonical Wnt signalling pathway, promoted the proliferation of LEPCs via downregulating the expression of SOX9 and upregulating SOX11 (unpublished data). Although it was reported that the nucleocytoplasmic shuttling of SOX9 and β-catenin might be crucial in the regulation of LEPC proliferation and differentiation (Menzel-Severing et al., 2018), its mechanism has not been thoroughly elucidated. This balance might be controlled by the ligands expressed in the mesenchymal cells, such as Dickkopf-2 (Walker et al., 2020), or the activation of ΔNp63 (Gouveia et al., 2019).

SOX9 is the marker of hair follicle stem cells. The cross-regulation between SOX9 and WNT signalling determines the specification and cell fate commitment of hair follicle stem cells (Xu et al., 2015). During hair formation, a Pcad^high^ placode cell undergoes asymmetric cell division in a WNT^high^ environment and generates two daughter cells: one WNT^low^SOX9^+^ and the other WNT^high^SOX9^-^. WNT^low^SOX9^+^ cells migrate towards the suprabasal layer and maintain stemness, while WNT^high^SOX9^-^ cells remain in the basal layer and undergo terminal differentiation (Ouspenskaia et al., 2016). The underlying mechanism of this cell specification lies in a WNT signalling gradient, in which WNT^low^SOX9^+^ cells respond to paracrine SHH expressed by WNT^high^SOX9^-^ cells. Moreover, the knockdown of SOX9 led to the dampened expression of Wnt signalling pathway genes, such as *LEF1*, *TCF1*, and *c-Myc* in goat hair follicle stem cells (He et al., 2018).

### 4.6 Other stem cells and progenitors

It was reported that in the salivary glands, the population of SOX9^+^ progenitor cells increases after radiation damage via the activation of the Wnt/β-catenin pathway (Xu et al., 2022). During the development of teeth, SOX9 and Wnt signalling regulate mesenchymal and epithelial interactions and control the expansion and differentiation of apical stem/progenitor cells (Lav et al., 2023). In addition, adipose mesenchymal-stem-cell-derived exosomes activate the Wnt/β-catenin pathway by upregulating the expression of SOX9, leading to accelerated proliferation and migration of human skin fibroblast cells and promoting skin wound healing (Qian et al., 2021).

In the embryonic neural stem cells (NSCs) of mice, NF-α1 inhibited the Wnt/β-catenin signalling pathway and repressed cell proliferation, while it activated the MAPK/MEK/Sox9 signalling pathway and promoted the differentiation of NSCs into astrocytes (Selvaraj et al., 2017). Notably, the promotive effect of NF-α1 on SOX9 preceded its inhibition on β-catenin. Nevertheless, whether NF-α1 indirectly inhibits β-catenin via SOX9 remains unclear.

## 5 Perspectives

Currently, the studies on the interactions of the SOX9-Wnt axis are mainly focused on the canonical Wnt signalling pathway. The mechanism between SOX9 and the noncanonical Wnt signalling pathway has not been fully elucidated. Moreover, it cannot be excluded that some interactions between SOX9 and β-catenin or GSK3β are regulated by non-Wnt signalling pathways because they might be modulated in a WNT-independent way (McDonald et al., 2012; Alanis et al., 2014).

Given the complexity of signalling pathways, it still remains to be answered how a three-dimensional dynamic network of SOX9-Wnt crosstalk is organized. The currently available data show us that nucleocytoplasmic shuttling and asymmetric division merit further study from a spatial perspective, whereas stage-specific regulations and bimodal effects are revelatory from a temporal perspective. In addition, the interactions between the SOX9-Wnt axis and other signalling pathways and molecules, such as Yap/Taz, need elucidation. Apart from the studies on molecular mechanisms, it is also important to elucidate the variations of SOX9-Wnt cross-regulations on different types of cells and tissues and to explore their correlations with cell functions from a spatiotemporal perspective in future work.

Lastly, novel treatments targeting the SOX9-Wnt axis have great potential in translational cell therapy to control cell proliferation, differentiation, and survival. For example, the manipulation of the SOX9-Wnt axis has been reported to control the culture conditions of *in-vitro*-expanded stem/progenitor cells, such as iPSC-derived insulin-producing cells (Xu et al., 2019) and LEPCs (Menzel-Severing et al., 2018), and regulate cell proliferation and differentiation. Our previous work also supports that SOX9-Wnt cross-regulation might be a potential tool to optimize *in vitro* cultivation of functional LEPC grafts for cell replacement therapy. Additionally, patients with diseases related to abnormal cell differentiation, such as diabetes mellitus that is caused by abnormal β-cells with ectopic expression of SOX9 and concomitant activation of Wnt signalling (Puri et al., 2013), may benefit from *in vivo* medication delivery targeting aberrant SOX9-Wnt cross-regulation.

In summary, although many studies have demonstrated that the cross-regulation between SOX9 and Wnt signalling pathway have potential in the clinical translation of stem cell therapy, its molecular mechanism needs further investigations to be fully addressed. A comprehensive recognition of the cross-talk network is indispensable for selecting a translational target with the highest safety and efficacy in the network, which are crucial for future *in vitro* and *in vivo* stem cell replacement therapy.

## 6 Conclusion

In conclusion, the cross-regulation between SOX9 and the canonical Wnt signalling pathway—through either mutual antagonism or activation—has been found to be involved in the physiological and pathological processes of stem/progenitor cells in different types of organs, tissues, anatomical locations, and stages of development. However, the underlying mechanisms of their mutual regulation in stem cells have not been fully elucidated and require further investigations.

## Abbreviations

APC, adenomatous polyposis coli; ARM, armadillo; CDK1, cyclin-dependent kinase 1; CKIα, casein kinase I alpha; CTD, C-terminal domain; DIM, dimerization domain; FZD, frizzled class receptors; GSK3β, glycogen synthase kinase 3 beta; HMG, high-mobility group; Hmga1, high-mobility group A1; ICAT, inhibitor of β-catenin and Tiff; ISC, intestinal stem cells; LEF, lymphoid enhancer factor; LEPC, limbal epithelial stem/progenitor cell; LRP, low density lipoprotein receptor-related protein; MAML2, mastermind-like transcriptional coactivator 2; Mast4; Microtubule-associated serine/threonine kinase 4; MSC, mesenchymal stem/progenitor cell; NCCs, neural crest cells; NES, nuclear export signal; NLS, nucleus localization signal; NSC, neural stem cell; NTD, N-terminal domain; ONFH, osteonecrosis of the femoral head; PQA, proline-glutamine-alanine; SOX9 △C, SOX9 C-terminal deleted mutant; SUMO, small ubiquitin-like modifier; TAC, C-terminal transactivation domain; TCF, T-cell factor; USP22, ubiquitin-specific peptidase 22.
